# Interparental conflict and adolescent online flaming: the chain mediating roles of psychological stress and online disinhibition

**DOI:** 10.3389/fpsyg.2026.1840562

**Published:** 2026-08-31

**Authors:** Suizhi Zeng, Zhijun Liu, Lei Zheng

**Affiliations:** 1 School of Psychology, Hainan Normal University, Haikou, China; 2 Hainan Provincial Research Base for Adolescent Psychological Development and Education, Hainan Normal University, Haikou, China; 3 Finance Office, Hainan Normal University, Haikou, China; 4 Finance Office, Qiongtai Normal University, Haikou, China

**Keywords:** benign online disinhibition, gender differences, interparental conflict, online flaming, psychological stress, toxic online disinhibition

## Abstract

**Objective:**

This study aimed to examine the relationship between interparental conflict and online flaming among Chinese adolescents, to test the mediating effects of psychological stress and online disinhibition (including toxic disinhibition and benign disinhibition) in this relationship, and to further explore gender differences in these mechanisms.

**Methods:**

Using convenience sampling, a cross-sectional survey was conducted with 1,096 adolescents, all of whom completed self-report measures of interparental conflict characteristics, online flaming, psychological stress, and online disinhibition. Based on the survey data, a chain mediation model was constructed and fitted through structural equation modeling, and gender differences in the overall model as well as in path coefficients were examined via multi-group analysis and pairwise comparisons.

**Results:**

Interparental conflict positively predicted adolescents' online flaming. Psychological stress, toxic online disinhibition, and benign online disinhibition each mediated this relationship. Furthermore, psychological stress and the two types of online disinhibition jointly formed two chain mediating pathways: “interparental conflict → psychological stress → toxic online disinhibition → online flaming” and “interparental conflict → psychological stress → benign online disinhibition → online flaming.” Although the overall hypothesized model showed no significant gender differences, both the direct effect of interparental conflict on psychological stress and the indirect effect of toxic online disinhibition in the interparental conflict–online flaming link were stronger for males than for females.

**Conclusion:**

This study reveals the underlying mechanisms through which interparental conflict contributes to adolescents' online flaming, indicating that intervening in interparental conflict, alleviating psychological stress, and reducing online disinhibition are effective ways to decrease such behavior.

## Introduction

With the pervasive penetration of the Internet, while facilitating global communication, it has also given rise to many negative phenomena, among which online flaming is particularly prominent (Hardaker, 2010; Craker and March, 2016). Online flaming is a form of online behavior in computer-mediated communication characterized by explicit hostility and aggression. It typically manifests as the unrestrained use of profanity, insults, curses, and condemnatory language, accompanied by intense emotional venting, with the intent to harm individuals or groups (Alonzo and Aiken, 2004; Willard, 2007; Jane, 2015; Andersen, 2021; Monge and Laurent, 2024). Its specific forms include malicious comments (Chen and Ng, 2017), online hostility (Yen et al., 2011), online aggressive speech (Cicchirillo et al., 2015), aggressive posts (Moore et al., 2012), online body shaming (Schlüter et al., 2023), online provocation (March and Marrington, 2019), and impolite comments (Rega et al., 2023), among others. However, these online misbehaviors may have numerous adverse effects on others and society. Victims may not only develop internalizing problems such as anxiety, sadness, suicidal behavior, and self-harm ideation (Niven et al., 2022; Thompson and Cover, 2022), but may also develop externalizing problems such as verbal aggression (Kwon and Gruzd, 2017), and may even be detrimental to social stability (Schäfer et al., 2022). Given these harms, elucidating the mechanisms of online flaming, especially among adolescents in the critical period of physical and mental development, has become an urgent topic with both theoretical and practical significance.

Drawing on social learning theory, some researchers have pointed out that adolescents chronically exposed to interparental conflict may come to view conflict as a normal way of solving problems and transfer this pattern to future interpersonal interactions, thereby increasing the risk of aggressive behavior (Bandura, 1977; Li et al., 2025). However, few studies have directly examined the relationship between interparental conflict and adolescents' online flaming from this theoretical perspective. Although some scholars have explored the effect of interparental conflict on adolescent cyberbullying (He et al., 2024), cyberbullying and online flaming differ considerably. Cyberbullying is typically defined as repeated, intentional aggression carried out through electronic media against victims who cannot easily defend themselves (Kowalski et al., 2014); it emphasizes the repetitiveness of aggression and encompasses forms such as online exclusion, online rumors, and online deception (Del Rey et al., 2015). In contrast, online flaming focuses more on a single, direct, hostile verbal outburst, does not usually target others, and primarily takes the form of written language (March and Marrington, 2019). This distinction suggests that the formation mechanisms of cyberbullying cannot simply be extrapolated to online flaming, and it is necessary to independently investigate the influence of interparental conflict on online flaming. However, the formation of online flaming may also involve mediating mechanisms. According to general strain theory, interparental conflict, as a significant negative stimulus, constitutes a typical source of stress for adolescents (Agnew, 2017). Interestingly, Wilson and Seigfried-Spellar (2023), based on general strain theory, found in their empirical study that the greater the perceived stress, the more online flaming behaviors individuals engage in. This suggests that psychological stress may serve as a mediating variable in the relationship between interparental conflict and online flaming. Furthermore, Zhang et al. (2022) drew on the strength model of self-control to examine the relationship between interparental conflict and self-control ability among Chinese adolescents. Their study found that prolonged exposure to interparental conflict continually depletes individuals' self-control resources, leading to a lack of self-control. For these adolescents, the anonymity, invisibility, and asynchronicity of cyberspace (Suler, 2004), their originally low inhibitory control is more easily triggered by the context, manifesting as a high level of online disinhibition—that is, a temporary suspension of offline social norms and inhibitory mechanisms. DeWall et al. (2011) pointed out that disinhibition in the online environment significantly promotes the occurrence of online flaming. Thus, online disinhibition may constitute another potential mediating variable linking interparental conflict and online flaming.

Notably, psychological stress and online disinhibition are not isolated. Recent research has shown that when adolescents are chronically exposed to stress, their limited self-control resources are continuously depleted, resulting in weakened behavioral inhibition (Ouyang and Shu, 2026). This suggests a possible positive association between psychological stress and online disinhibition, and that the two may serve as chain mediators in the relationship between interparental conflict and online flaming. However, no study has simultaneously examined the parallel and chain mediating mechanisms of psychological stress and online disinhibition between them. Therefore, the present study integrates social learning theory, general strain theory, and the strength model of self-control to construct a chain mediation model, aiming to systematically reveal the mediating mechanisms of psychological stress and online disinhibition in the relationship between interparental conflict and online flaming. In addition, considering that males and females may exhibit different psychological and behavioral responses to the same stimuli as shaped by sociocultural factors (Oransky and Fisher, 2009; Eagly and Wood, 2012), this study will also explore gender differences in the overall mediation model or specific pathways, to reveal whether the mechanisms through which interparental conflict influences online flaming vary by gender. This model contributes to a comprehensive analysis of the combined effects of interparental conflict, psychological stress, online disinhibition, and gender on online flaming, and holds significant practical value for reducing online flaming and promoting positive mental health development among secondary school students.

### The relationship between interparental conflict and online flaming

Interparental conflict refers to verbal or physical disputes and aggression between parents arising from disagreements or other causes, and is typically defined along dimensions such as frequency, intensity, content, style, and resolution status (Chi and Xin, 2003). A large body of research has demonstrated that interparental conflict is closely associated with a variety of adjustment difficulties in children (Tanaka et al., 2010; Koçak et al., 2017). Social learning theory (Bandura, 1978) posits that individuals acquire behavioral patterns by observing significant models and reproduce them in corresponding situations. Accordingly, adolescents who are chronically exposed to hostile, insulting, and dysregulated verbal exchanges in interparental conflict are likely to internalize such verbal aggression as a habitual mode of expressing discontent, and subsequently transfer it to their own interpersonal interactions. Ample direct evidence indicates that perceived interparental conflict increases adolescents' offline aggression (Chu et al., 2025; Li et al., 2025). In contrast, research on certain specific forms of online aggression, such as online flaming, remains limited. The online environment is more complex than the offline context, and the forms of aggression that adolescents can exhibit are increasingly diverse. Existing research has largely concentrated on cyberbullying and has confirmed that interparental conflict positively predicts cyberbullying (He et al., 2023, 2024; Wang et al., 2026); however, investigations into online flaming are notably scarce. Online flaming is characterized by a single, direct hostile verbal outburst, typically does not target a specific individual, and primarily takes the form of written language (Li et al., 2008; March and Marrington, 2019). Cyberbullying, by contrast, emphasizes repetition, does not require direct confrontation, often targets a specific victim, and encompasses a wider variety of aggressive forms (Del Rey et al., 2015). These distinctions indicate that the formation mechanisms underlying cyberbullying cannot simply be extrapolated to online flaming; the latter merits independent investigation. Nevertheless, the transfer of aggressive patterns predicted by social learning theory is not negated by differences in specific forms: the aggressive behaviors modeled in interparental conflict can still provide readily available scripts for online flaming. Based on this reasoning, the aggressive tendencies shaped by interparental conflict can extend to the online context and manifest as online flaming. Accordingly, we propose Hypothesis 1:

H1: Interparental conflict will positively predict adolescents' online flaming.

### The mediating role of psychological stress

Psychological stress refers to the psychological distress or perceived threat triggered by various stressful events and adverse living environments, manifesting as physical and mental tension and unease (Yang and Huang, 2003). General strain theory posits a direct association between strain and maladaptive behaviors; when strain exceeds a certain threshold, a cumulative stress effect emerges, which in turn fosters such behaviors (Agnew, 1992). A Chinese study confirmed this theory, finding that adolescents with high stress levels engage in online flaming to attack others as a means of relieving life stress (Yin and Xuan, 2023). Moreover, a large body of research has now established that adolescents' perceived stress is significantly and positively correlated with both their online and offline aggressive behavior (Wei et al., 2022; Yang et al., 2022; Yin et al., 2025). Notably, general strain theory indicates that negative stimuli are an important source of individual strain, with interparental conflict being one of the important sources (Agnew, 2017). Longitudinal studies on adolescents have demonstrated that higher perceived interparental conflict at an earlier stage predicts higher stress levels at a subsequent stage (Heinze et al., 2020). Synthesizing the above theories and evidence, it can be inferred that interparental conflict, as a negative stimulus, elevates adolescents' psychological stress, and that to relieve this continuously accumulated tension, they may turn to online flaming. Accordingly, we propose Hypothesis 2:

H2: Psychological stress mediates the relationship between interparental conflict and adolescents' online flaming.

### The mediating role of online disinhibition

Online disinhibition refers to the reduced behavioral inhibition individuals show in computer-mediated communication (Lapidot-Lefler and Barak, 2012), where people act in ways that deviate from their offline conduct—for instance, discreet individuals may forward private emails or gossip online (Joinson, 1998). It takes two forms: benign online disinhibition and toxic online disinhibition. Benign online disinhibition involves pro-social acts such as sharing personal secrets, expressing wishes, or showing kindness and generosity; toxic online disinhibition, by contrast, entails detrimental activities like threats, bullying, and verbal abuse (Suler, 2004). Moreover, the phenomenon encompasses six features: dissociative anonymity, invisibility, asynchronicity, solipsistic introjection, dissociative imagination, and minimization of authority (Suler, 2004). Among these, the minimization of authority weakens its role online, leading individuals to speak more freely and engage in inappropriate behaviors (Suler, 2004). Research has indicated that anonymity contributed to increased flaming (Nitschinsk et al., 2022) and self-disclosure (Clark-Gordon et al., 2019), and found that higher online disinhibition positively predicts inflammatory, provocative, and hateful comments (Wu et al., 2022). The lack of nonverbal feedback online encourages more spontaneous and sometimes aggressive self-expression. Furthermore, online disinhibition is associated with a more intense expression of negative emotions in digital environments (Samsidar, 2025). Few studies have separately examined the effects of toxic and benign online disinhibition on online flaming; most studies have focused on their relationship with cyberbullying. For example, a study by Japanese researchers found that both toxic and benign online disinhibition positively predicted cyberbullying (Udris, 2014). In contrast, Chinese scholars found that toxic online disinhibition positively predicted cyberbullying, whereas benign online disinhibition did not (Chu et al., 2023). The boundary between benign and toxic online disinhibition is sometimes ambiguous: some hostile remarks may convey positive meanings, while excessive self-disclosure can be harmful (Suler, 2004). It is therefore plausible that benign disinhibition may trigger or escalate into aggressive behavior. Moreover, given that online flaming and cyberbullying share common features of aggressive expression, both forms of disinhibition may predict online flaming.

Notably, interparental conflict may contribute to higher levels of online disinhibition among adolescents. Chronic interparental conflict may lead adolescents to accumulate negative emotions and feel unsupported (Yang and Liu, 2026), driving them to use social media to alleviate distress and fulfill unmet needs (Dou et al., 2025). However, these teenagers who enter cyberspace may engage in behaviors that they would normally keep under control in most circumstances. For example, heightened insulting and provocative behavior online may reflect toxic disinhibition, whereas a stronger tendency to seek comfort and support online may reflect benign disinhibition. Furthermore, according to the emotional security hypothesis, interparental conflict engenders emotional insecurity and negative affect, which, as posited by the strength model of self-control, deplete limited self-regulatory resources and thereby diminish online inhibition (Davies and Cummings, 1994; Baumeister et al., 2007). Research has shown that negative emotions consume substantial self-control resources (Chester et al., 2016). This suggests that interparental conflict may deplete inhibitory resources, heightening the tendency toward disinhibition. In light of this, online disinhibition may mediate the relationship between interparental conflict and online flaming. However, few studies have examined this mediating mechanism, particularly the distinct roles of benign and toxic online disinhibition. Therefore, we propose Hypothesis 3:

H3: Online disinhibition (benign and toxic disinhibition) mediates the association between interparental conflict and adolescents' online flaming.

### Chain mediating role of psychological stress and online disinhibition

Based on the literature reviewed above, psychological stress and online disinhibition may both act as significant mediators between interparental conflict and online flaming. Notably, adolescents under high life stress tend to seek relief online. Given the anonymity and invisibility of the Internet, they are more inclined than in offline contexts to relieve stress through aggressive acts (toxic online disinhibition) or through seeking social support (benign online disinhibition) (Chu et al., 2023). Second, according to the strength model of self-control (Baumeister et al., 2007), stress diminishes cognitive resources, resulting in lower self-control and increased disinhibition tendencies (Soysa and Gardner, 2013). A longitudinal study in the United Kingdom indicated that early life stress predicts future disinhibited behavior in children (Enoch et al., 2010). Moreover, Chu et al. (2023) observed that individuals, when confronted with difficult events, tend to suppress their emotions offline to conform to social norms, whereas online, they engage in disinhibited thinking and express themselves without restraint. These studies demonstrate that psychological stress may contribute to online disinhibition. Accordingly, we posit Hypothesis 4:

H4: Psychological stress and online disinhibition (benign and toxic disinhibition) sequentially mediate the relationship between interparental conflict and adolescents' online flaming.

### Gender differences in structural equation modeling

Gender, defined as the activity of managing situated conduct in line with normative expectations for one's sex category, both emerges from and reinforces claims to membership in that category (West and Zimmerman, 1987). According to male gender role norms, dominance and aggression are important elements, rendering males more vulnerable to various adjustment problems (Oransky and Fisher, 2009). In contrast, social role theory posits that females are more inclined to form and maintain close relationships, express emotions, provide support, and emphasize harmony and cooperation rather than dominance and control in group interactions (Eagly and Wood, 2012). Thus, gender is likely a key factor in the generative mechanism of online flaming. In the context of family stress, Erel and Burman (1995) noted that, compared to girls, boys exposed to parental conflict experience more intense stress and learn more aggressive behaviors. Similarly, Zhang et al. (2022) found that witnessing interparental violence significantly predicted cyberbullying among Chinese adolescents, with boys exhibiting more such behaviors than girls. Luo et al. (2023) further revealed that the effect of stress on offline bullying varied by gender, with males being more likely to engage in offline bullying. However, findings regarding cyberbullying are inconsistent: Chu et al. (2023) showed that the direct effects of stress on cyberbullying and on toxic online disinhibition did not differ significantly by gender, yet the pathway from toxic online disinhibition to cyberbullying was significant only for males. Echoing this pattern, Huang et al. (2020) reported that males perceived significantly stronger toxic online disinhibition than females and were more likely to engage in cyberbullying, whereas no gender difference emerged in benign online disinhibition. In summary, existing research has largely focused on gender differences in the mechanisms underlying cyberbullying, whereas the exploration of online flaming remains relatively scarce. Given that both cyberbullying and online flaming are essentially forms of online flaming, we hypothesize that the overall mediation model—or certain paths within it—may differ by gender. Accordingly, we plan to test this hypothesis using multi-group analysis and pairwise comparisons.

**Figure 1:**
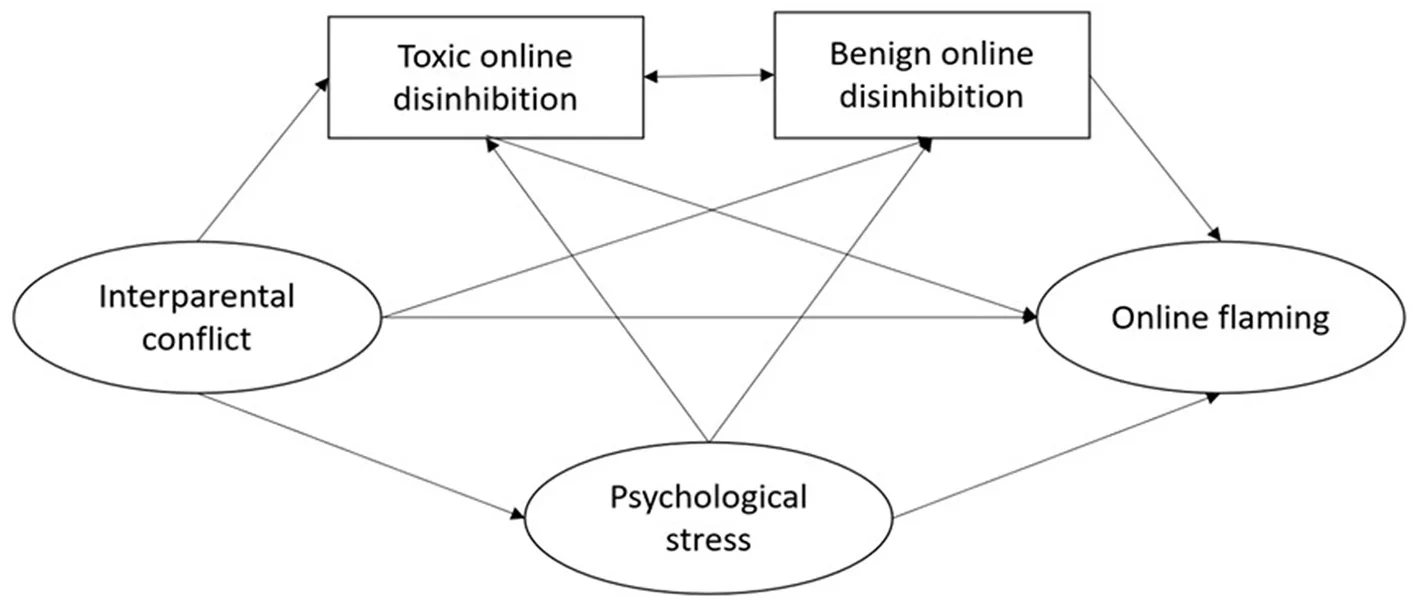
Chained mediation model.

This study develops a serial mediation model (Figure 1) grounded in the social learning theory, general strain theory, and the strength model of self-control to investigate the mediating effects of psychological stress and online disinhibition between interparental conflict and online flaming.

## Methods

### Participants

Before data collection, an a priori power analysis was conducted using G^*^Power for a multiple regression with six predictors, specifying a two-tailed α = 0.05, power = 0.80, and an effect size *f* ^2^ = 0.15 (Faul et al., 2009). The result indicated that a minimum of 146 participants was required. Based on this requirement, we employed convenience sampling to distribute 1,208 questionnaires among students in Grades 7 to 9 from three secondary schools in Haikou, and 1,203 were returned. After excluding questionnaires with more than 10% incomplete responses and those exhibiting patterned responding, 1,096 valid questionnaires were retained, yielding a valid response rate of 90.73%. The final sample ranged in age from 12 to 15 years (*M* = 13.55, SD = 0.95) and comprised 528 boys (48.2%) and 568 girls (51.8%). By grade, 371 were in Grade 7 (33.9%), 355 in Grade 8 (32.3%), and 370 in Grade 9 (33.8%). Regarding residence, 653 participants lived in urban areas (59.6%) and 443 in rural areas (40.4%). In terms of sibling status, 652 were only children (59.5%), and 444 were non-only children (40.5%). This study was approved by the ethics committee of the corresponding author's institution. Before the survey administration, permission from parents and teachers, as well as informed consent from the participants, were obtained. The questionnaires were administered uniformly as part of the regular mental health education curriculum. The administrator read the instructions aloud, explained relevant study information—including the commitment to data confidentiality and anonymity—and explicitly informed participants that participation was entirely voluntary and that they could withdraw at any time.

### Measurements

#### Interparental conflict characteristics scale

The Chinese adaptation of the Children's Perception of Interparental Conflict Scale (CPIC), first developed by Grych et al. (1992) and subsequently amended by Chi and Xin (2003), was utilized. This study employed the Conflict Characteristics Scale, which comprises three dimensions—conflict intensity, conflict frequency, and conflict resolution—with a total of 16 items. A four-point Likert scale was employed, spanning from 1 (strongly disagree) to 4 (strongly agree), and the values for each dimension were aggregated to derive a total interparental conflict score. Elevated scores on this scale signify increased levels of interparental conflict. The Cronbach's alpha coefficient for this scale in the present investigation was 0.933.

#### Online flaming scale

The Online Flaming Scale, developed by Li (2008), was utilized. The scale comprises four dimensions—aggression, irritation, hostility, and conflict—totaling 20 items. A five-point Likert scale was employed, with ratings spanning from 1 (not at all) to 5 (all the time), and the scores for each dimension were aggregated to derive a total score for online flaming. An elevated score on this measure signifies an increased degree of online flaming. The Cronbach's alpha coefficient for this scale in the study was 0.936.

#### Online disinhibition scale

The Online Disinhibition Scale was developed by Udris (2014) and has strong reliability and validity among the sample of junior high school students in China (Zhang et al., 2019). The scale comprises two dimensions—benign online disinhibition and toxic online disinhibition—with a total of 11 items. A four-point Likert scale was employed, with scores from 1 (strongly disagree) to 4 (strongly agree). The scores of the dimensional items were aggregated to derive the total scores for benign and toxic online disinhibition, respectively, with higher scores indicating greater levels of both types of online disinhibition. This study reported Cronbach's alpha scores of 0.847 for the benign online disinhibition scale and 0.731 for the toxic online disinhibition scale.

#### Psychological stress scale

The English version of the Psychological Stress Scale was developed by Cohen et al. (1983), while the Chinese adaptation was modified by Yang and Huang (2003). The scale comprises two dimensions—tension and loss of control—totaling 14 items. The scoring utilizes a five-point Likert scale, ranging from 1 point for “never” to 5 points for “always.” A higher score correlates with increased psychological stress in the subject. The Cronbach's alpha for this scale in the study was 0.776.

### Data analysis

We used R 4.5.1 as an analysis tool and took RStudio/2024.04.2+764 as an integrated development environment (IDE), and further analyzed the data. The R package psych was utilized to conduct reliability analysis (calculating Cronbach's alpha for each scale), evaluate common method bias (controlling for the unmeasured latent method factor), and calculate descriptive statistics (including means, standard deviations, and correlation coefficients) (Revelle, 2026). The R package lavaan, which can apply the maximum likelihood method for model estimation and evaluation, was employed to execute confirmatory factor analysis (CFA), assess the mediating effects within structural equation modeling (SEM) when using bootstrap resampling, and carry out pairwise comparisons, examining gender differences in the pathways (Rosseel et al., 2025). Multi-group analyses were conducted utilizing the R packages lavaan, lavaan.mi, and semTools to examine the gender differences in the overall model (Jorgensen et al., 2026).

## Results

### The results of descriptive statistics and correlation analysis of variables

Table 1 presents the outcomes of the descriptive statistics and correlation analysis of the variables. The correlation study indicates a substantial positive association between interparental conflict, psychological stress, benign online disinhibition, toxic online disinhibition, and online flaming.

**Table 1:** Descriptive statistics and correlation analysis.

Variables	*M* ±SD	1	2	3	4	5	6	7
1. Gender	–	1						
2. Age	13.49 ± 0.95	0.07^*^	1					
3. IC	2.01 ± 0.61	0.03	0.14^**^	1				
4.PS	2.95 ± 0.56	0.00	0.20^**^	0.66^**^	1			
5. BD	2.17 ± 0.60	0.16^**^	0.15^**^	0.43^**^	0.47^**^	1		
6. TD	1.22 ± 0.53	0.18^**^	0.20^**^	0.43^**^	0.40^**^	0.41^**^	1	
7. OF	1.82 ± 0.85	0.18^**^	0.19^**^	0.64^**^	0.66^**^	0.68^**^	0.69^**^	1

Boy = 1, Girl = 0; IC, interparental conflict; PS, psychological stress; BD, benign online disinhibition; TD, toxic online disinhibition; OF, online flaming;

*p < 0.05,

**p < 0.01.

### Common method bias test

The common method bias test was performed by employing the “controlling for an unmeasured latent method factor” approach. We developed a one-factor model (M1) comprising solely the trait factors, followed by a two-factor model (M2) that included both the trait factors and a method factor. A comparison of M1 and M2 revealed the following changes in fit indices: ΔCFI = 0.021, ΔTLI = 0.017, and ΔRMSEA = 0.003. Following the incorporation of the method factor, the increases in the CFI and TLI were less than 0.1, and the reduction in the RMSEA was below 0.05, suggesting that the model fit does not exhibit significant enhancement post-addition of the method factor, thereby indicating that the common method bias in this study is not substantial (Wen et al., 2018).

### Measurement model

Initially, confirmatory factor analysis indicated that all measurement models demonstrated a satisfactory fit. According to Wu and Weng (2011), items related to interparental conflict and psychological stress were each distributed into three parcels to function as three observed variables for the latent variables. The second-order latent variable for online flaming was represented by four first-order latent variables: aggression, irritability, hostility, and conflict, each of which was measured using three item parcels. Table 2 displays the factor loadings, composite reliability (CR), and average variance extracted (AVE) from the measurement model. The standardized factor loadings for all observed variables ranged from 0.62 to 0.98, achieving significance at the *p* < 0.001 level; composite reliability varied from 0.60 to 0.95; and the average variance extracted spanned from 0.53 to 0.85. The overall measurement model fit was deemed good (χ^2^/df = 3.66, CFI = 0.96, TLI = 0.95, and RMSEA = 0.049), making it appropriate for structural equation modeling.

**Table 2:** Results of the measurement model.

Latent variable	Observed variable	Est./S.E.	β	CR	AVE	*p*
Interparental conflict	CF	31.50	0.86	0.83	0.63	<0.001
	CI	28.76	0.76			<0.001
	CR	26.67	0.75			<0.001
Psychological stress	PS1	24.53	0.71	0.82	0.60	<0.001
	PS2	29.61	0.85			<0.001
	PS3	26.10	0.76			<0.001
Aggression	AG1	15.84	0.62	0.78	0.55	<0.001
	AG2	18.30	0.81			<0.001
	AG3	18.21	0.78			<0.001
Irritability	IR1	3.35	0.77	0.82	0.60	<0.001
	IR2	3.35	0.78			<0.001
	IR3	3.35	0.78			<0.001
Hostility	HO1	9.94	0.71	0.77	0.53	<0.001
	HO2	9.99	0.74			<0.001
	HO3	9.98	0.74			<0.001
Conflict	CO1	14.83	0.69	0.79	0.56	<0.001
	CO2	15.41	0.76			<0.001
	CO3	15.56	0.80			<0.001
Online flaming	AG	15.23	0.85	0.95	0.83	<0.001
	IR	3.27	0.98			<0.001
	HO	9.32	0.93			<0.001
	CO	13.57	0.89			<0.001

CF, conflict frequency; CI, conflict; CR, conflict resolution; PS, psychological stress; AG, aggression; IR, irritability; HO, hostility; CO, conflict.

### Analysis of mediated effects

Initially, structural equation modeling was employed to examine the process by which interparental conflict influences middle school children's online flaming, utilizing the maximum likelihood method for model estimation and evaluation. The results indicated that the fit indices (χ^2^/df = 3.52, CFI = 0.96, TLI = 0.95, and RMSEA = 0.048) satisfied the criteria, signifying a good model fit. In R, employing the nonparametric percentile Bootstrap method, the mediation test was conducted with 5,000 bootstrap resamples; if the 95% confidence intervals of these indirect effects excluded 0, this signified that the mediation effect was significant.

**Figure 2:**
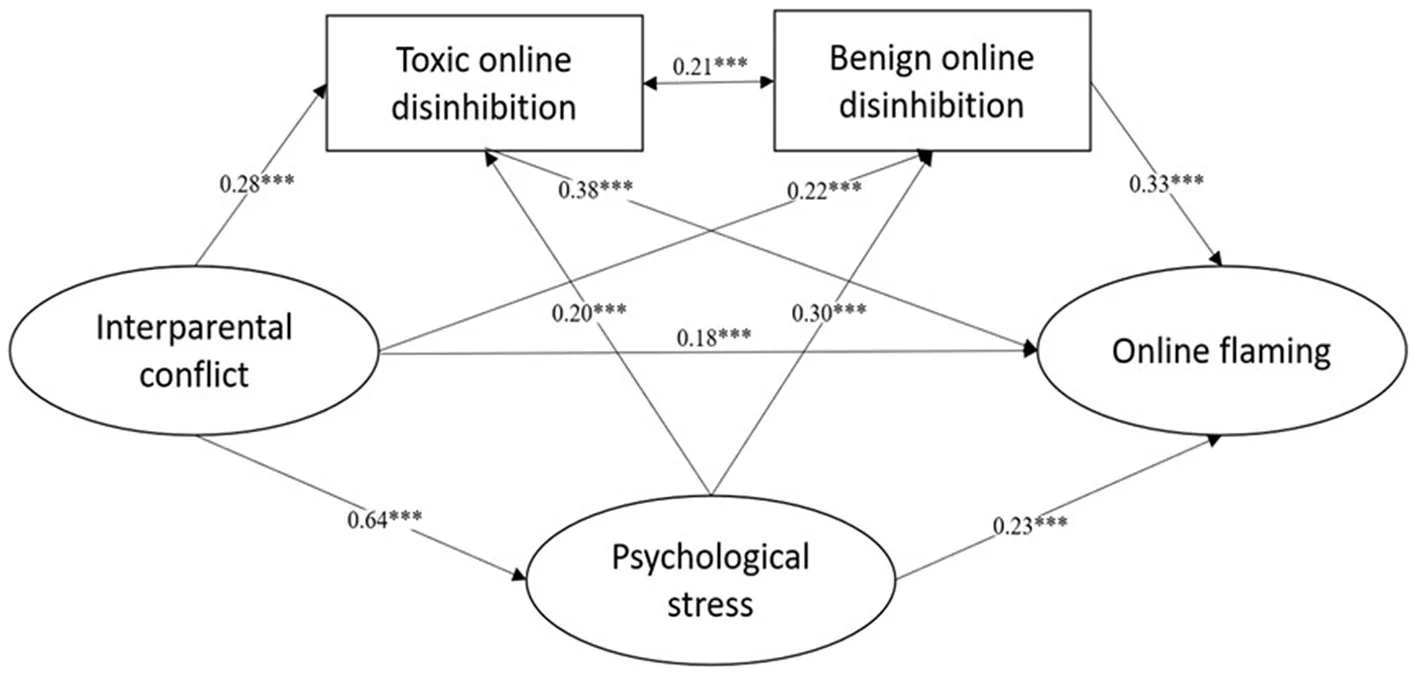
Standardized path coefficients of the chained mediation model with the full sample. The coefficients of the covariates, including gender and age,were not shown in the figure. In this figure, ^***^ *p* < 0.001.

Secondly, Table 3 displays the outcomes of the structural equation modeling. The standardized path coefficients among the main variables are illustrated in Figure 2 for improved clarity. The findings demonstrated that interparental conflict was a significant predictor of psychological stress (β = 0.64, *p* < 0.001), toxic online disinhibition (β = 0.28, *p* < 0.001), benign online disinhibition (β = 0.22, *p* < 0.001), and online flaming (β = 0.18, *p* < 0.001). Moreover, psychological stress was a positive predictor of toxic online disinhibition (β = 0.20, *p* < 0.001), benign online disinhibition (β = 0.30, *p* < 0.001), and online flaming (β = 0.23, *p* < 0.001). Toxic (β = 0.38, *p* < 0.001) and benign (β = 0.33, *p* < 0.001) online disinhibition were significant positive predictors of online flaming.

**Table 3:** Structural equation model regression results.

Independent variables	Outcome variables	β	*z*	*p*
Age	Psychological stress	0.64	16.12	<0.001
Gender		0.13	4.63	<0.001
Interparental conflict		−0.01	−0.30	0.763
Age	Toxic online disinhibition	0.28	6.32	<0.001
Gender		0.11	3.95	<0.001
Psychological stress		0.17	6.27	<0.001
Interparental conflict		0.20	3.98	<0.001
Age	Benign online disinhibition	0.22	4.72	<0.001
Gender		0.05	1.80	0.073
Psychological stress		0.15	5.54	<0.001
Interparental conflict		0.30	6.54	<0.001
Age	Online flaming	0.18	4.43	<0.001
Gender		0.00	−0.06	0.955
Psychological stress		0.05	2.59	<0.010
Toxic online disinhibition		0.23	5.87	<0.001
Benign online disinhibition		0.38	11.30	<0.001
Interparental conflict		0.33	10.67	<0.001

Table 4 displays the total, direct, and indirect effects together with 95% confidence intervals derived from the structural equation modeling. The study investigated the mediating effect of psychological stress on the link between interparental conflict and online flaming, revealing that psychological stress significantly mediated this relationship (β = 0.15, *p* < 0.001). The mediating effects of toxic online disinhibition (β = 0.11, *p* < 0.001) and benign online disinhibition (β = 0.07, *p* < 0.001) on the association between interparental conflict and online flaming were significant. The chain-mediated effects were identified between interparental conflict and online flaming via psychological stress and toxic online disinhibition (β = 0.05, *p* < 0.001), as well as through psychological stress and benign online disinhibition (β = 0.06, *p* < 0.001).

**Table 4:** Total, direct, and indirect effects of the structural equation model.

Effect	Path	β	95%CI		Effect ratio
			Lower	Upper	
Direct effect	IC → OF	0.18	0.10	0.26	0.29
Indirect effect	IC → PS → OF	0.15	0.10	0.20	0.24
	IC → TD → OF	0.11	0.07	0.14	0.18
	IC → BD → OF	0.07	0.04	0.11	0.11
	IC → PS → TD → OF	0.05	0.03	0.08	0.08
	IC → PS → BD → OF	0.06	0.04	0.09	0.10
Total effect	IC → OF	0.62			

5,000 bootstrap samples. IC, interparental conflict, OF, online flaming, PS, psychological stress, TD, toxic online disinhibition, BD, benign online disinhibition.

### Multiple-group analysis and pairwise comparisons

**Figure 3:**
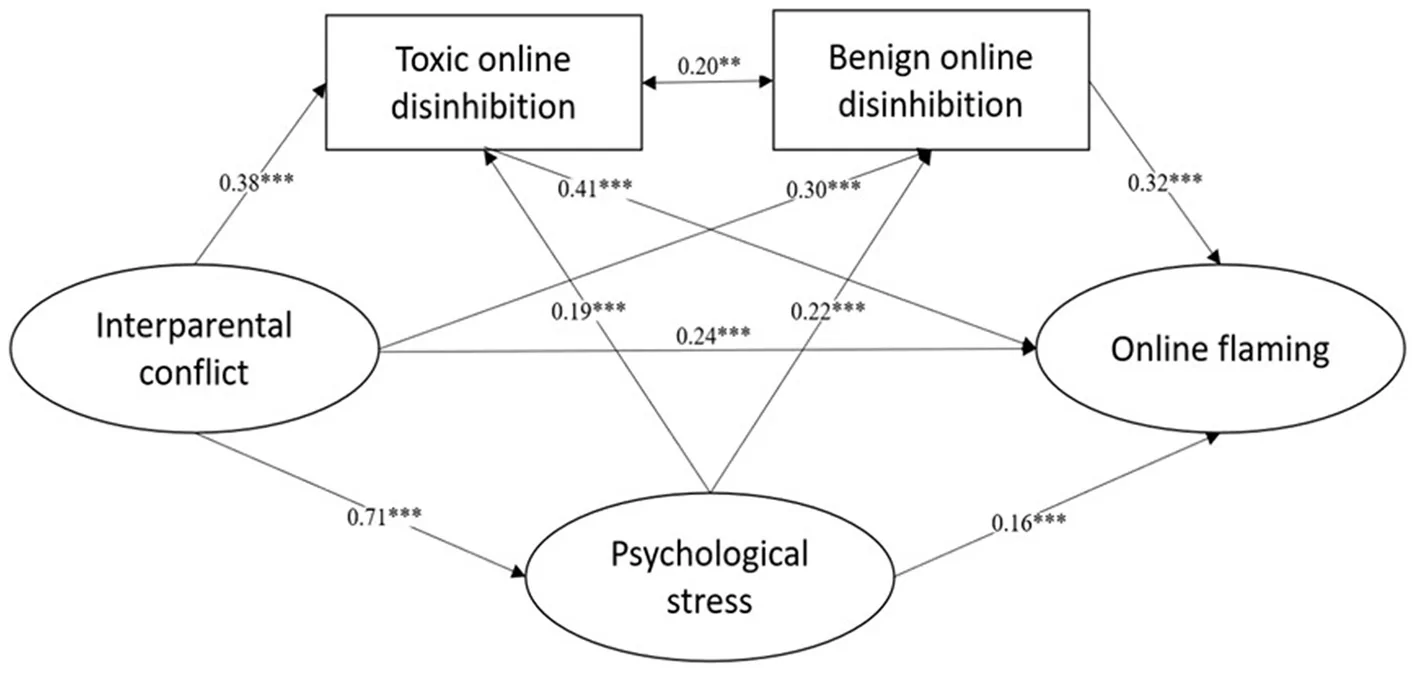
Standardized path coefficients of the chained mediation model with male subgroups. The coefficients of the covariate age were not shown in the figure, **p < 0.01, ***p < 0.001.

**Figure 4:**
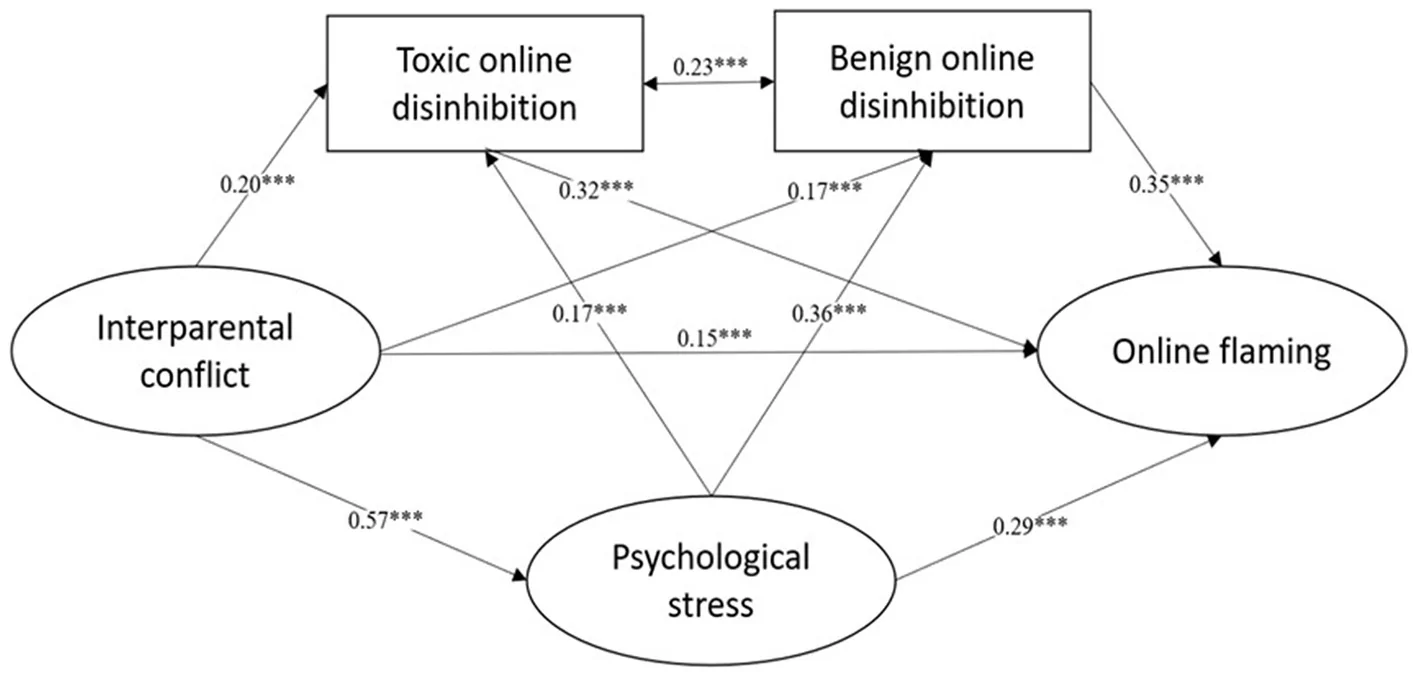
Standardized path coefficients of the chained mediation model with female subgroups. The coefficients of the covariate age were not shown in the figure, ***p < 0.001.

Initially, the structural equation model was developed separately for male and female groups to assess model fit. If the theoretical model fit satisfied the criteria, a multi-group analysis was subsequently conducted. The findings indicated that the model fit for males (χ^2^/df = 2.61, CFI = 0.95, TLI = 0.94, and RMSEA = 0.06) and females (χ^2^/df = 2.38, CFI = 0.95, TLI = 0.94, and RMSEA = 0.05) satisfied the recommended criteria for multi-group comparisons. The structural equation models for male and female groups are illustrated in Figures 3, 4, respectively.

Secondly, given that the fit indices of the structural equation models for both genders met the requirements for multi-group comparison, we specified an unconstrained model (M, serving as the baseline) and a constrained model (M1) in which all structural weights were set equal across genders. The baseline model demonstrated good fit (χ^2^/df = 2.45, CFI = 0.95, TLI = 0.94, and RMSEA = 0.052). A chi-square difference test indicated that M1 fit significantly worse than *M* (Δχ^2^ = 73.30, *p* < 0.01), suggesting that path coefficients might differ across genders. However, given the large sample size (*N* > 1000), the chi-square test is prone to excessive sensitivity; therefore, changes in fit indices were further examined. The results showed that |ΔCFI| = 0.005 (< 0.010) and ΔRMSEA = 0.001 (< 0.015), neither exceeding the critical thresholds, indicating that the overall model structure was invariant across genders (Chen, 2007).

On this basis, to probe gender differences in specific paths, we first conducted pairwise comparisons of the indirect effects using the bootstrap method (5,000 resamples) and applied the Benjamini–Hochberg correction to the *p*-values (Benjamini and Hochberg, 1995). As shown in Table 5, only the indirect effect of “interparental conflict → toxic online disinhibition → online flaming” reached marginal significance (Δβ = 0.10, *p* = 0.06), hinting at a trend for this indirect effect to be slightly stronger in males than in females. Subsequently, we compared all direct paths across genders (Table 6) and found that only the path from interparental conflict to psychological stress exhibited a significant gender difference (Δβ = 0.14, *p* = 0.05), with a larger coefficient for males (β = 0.71, *p* < 0.001) than for females (β = 0.57, *p* < 0.001); all remaining direct paths showed no significant differences.

**Table 5:** Pairwise comparison of simple and chained mediating effects across genders.

Effect	Path	Δβ	95%CI		*p*	Adjusted *p*
			Lower	Upper		
Simple mediation	IC → PS → OF	−0.06	−0.16	0.04	0.34	0.37
	IC → TD → OF	0.10	0.02	0.17	< 0.05	0.06
	IC → BD → OF	0.04	−0.03	0.10	0.26	0.37
Chained mediation	IC → PS → TD → OF	0.03	−0.03	0.08	0.37	0.37
	IC → PS → BD → OF	−0.02	−0.07	0.03	0.35	0.37

**Table 6:** Pairwise comparison of direct effects across genders.

Independent variables	Outcome variables	Δβ	*z*	*p*	Adjusted *p*
Interparental conflict	Psychological stress	0.14	−2.89	< 0.01	0.05
Age		−0.01	1.12	0.26	0.38
Interparental conflict	Toxic online disinhibition	0.19	−2.18	< 0.05	0.11
Age		0.11	−1.91	0.06	0.15
Psychological stress		0.02	−0.22	0.82	0.82
Interparental conflict	Benign online disinhibition	0.13	−1.50	0.13	0.22
Age		0.03	−0.56	0.58	0.68
Psychological stress		−0.14	1.64	0.10	0.22
Interparental conflict	Online flaming	0.10	−1.52	0.13	0.22
Age		0.02	−0.39	0.69	0.75
Psychological stress		−0.13	2.12	< 0.05	0.11
Toxic online disinhibition		0.10	−2.19	< 0.05	0.11
Benign online disinhibition		−0.03	0.61	0.54	0.68

Author queries.

## Discussion

### The direct effect of interparental conflict on online flaming

The findings supported Hypothesis 1, aligning with previous studies (He et al., 2023; Wang et al., 2026), and demonstrated a significant direct effect of interparental conflict on online flaming among junior high school students. In line with social learning theory (Bandura, 1978), adolescents may acquire maladaptive behavioral patterns—such as verbal insults, physical aggression, and oppositional behaviors—through observing negative parental interactions, and they may adopt these behaviors as problem-solving strategies (Wang et al., 2026). Consequently, when interacting in cyberspace, these adolescents are likely to act out aggressive scripts and express negative affect learned at home, which in turn manifests as online flaming.

### The mediating role of psychological stress

Psychological stress was identified as a mediator in the association between interparental conflict and online flaming. Specifically, interparental conflict contributes to psychological stress, thereby leading junior high school students to engage in online flaming. This finding corroborates Hypothesis 2 and aligns with a prior study (Jang et al., 2014). The findings are also consistent with general strain theory (Agnew, 1992). When individuals encounter interparental conflict, they experience significant emotional distress (Kuhlman et al., 2018). As the stress endured by adolescents accumulates (Liu and Liu, 2021), they may seek to alleviate this pressure, and aggression toward others may serve as a form of release (Mifune et al., 2017). Moreover, the anonymity inherent in online communication reduces the consequences associated with undesirable behaviors. This leads some individuals to perceive cyberspace as an optimal outlet for emotional stress, which results in online flaming, such as aggression and verbal abuse (Piko et al., 2017).

### The mediating role of online disinhibition

The study confirmed that both benign online disinhibition and toxic online disinhibition mediated the effect of interparental conflict on online flaming among junior high school students, thereby supporting Hypothesis 3. In the first stage of the mediation pathway, interparental conflict positively predicted the level of online disinhibition, which is consistent with the Emotional Security Hypothesis and the Strength Model of Self-Control (Davies and Cummings, 1994; Baumeister et al., 2007). Specifically, interparental conflict tends to provoke excessive emotional reactions such as anger and sadness in children (Zhou et al., 2017); to alleviate these negative emotions, adolescents are inclined to retreat into the relatively safe online space. At the same time, negative emotions consume substantial psychological resources, leading to ego depletion and weakened inhibitory control, which in turn further elevate online disinhibition. Consequently, both forms of disinhibition positively predicted online flaming. Toxic online disinhibition refers to the tendency to engage in problematic behaviors in cyberspace (Suler, 2004), and the present study found that it fosters online flaming, similar to previous research (Chu et al., 2023). Immersed in the anonymity and weak constraints of the virtual environment (Scott et al., 2022), adolescents with high toxic disinhibition often perceive themselves as unaccountable for their actions and thus freely resort to abusive and aggressive online behaviors. In contrast, benign online disinhibition is characterized by a greater willingness to disclose hidden emotions, fears, and wishes and to express kindness and help others (Suler, 2004). However, this study also found that it contributes to online flaming (Udris, 2014). As Suler (2004) noted, the boundary between benign and toxic disinhibition is sometimes blurred and subject to group norms; hence, there are intrinsic mechanisms through which benign disinhibition drives online flaming. First, after joining online communities of interest, some adolescents' views become increasingly polarized under the influence of group interaction and peer pressure (Dong et al., 2026; Sun et al., 2026). When their own views or their community is disparaged, they respond with verbal attacks (Yang and Zeng, 2024) and construct such attacks as positive acts of defending their community and self-concept. This benign cognitive orientation lowers the threshold for retaliation and grants self-permission for aggression through moral justification. Second, individuals who experience verbal abuse online often retaliate in kind (Meriläinen and Ruotsalainen, 2024) and perceive such counterattacks as morally positive behaviors. Finally, adolescents who perpetrate online flaming may position themselves as agents of justice, viewing the shaming or attacking of others' misconduct as legitimate punishment (Wang and Yun, 2023; Ge, 2020). In the eyes of these adolescents, flaming is imbued with prosocial or moral positive meaning, thereby enabling benign disinhibition to be transformed into actual attacks.

### The serial mediating role of psychological stress and online disinhibition

The serial mediating role of psychological stress and online disinhibition was examined. Ultimately, psychological stress and online disinhibition (both benign and toxic) were identified as serial mediators linking interparental conflict to online flaming, supporting Hypothesis 4. As a significant stressor, interparental conflict induces psychological stress (Heinze et al., 2020), which exhausts an individual's finite self-control resources (Baumeister et al., 2007). As these resources are depleted through self-regulatory efforts, the capacity for self-control diminishes, and ongoing psychological stress further erodes psychological resources. Notably, adolescents suffering from elevated psychological stress often perceive cyberspace as a safe location to relieve their stress (Yin and Xuan, 2023). Consequently, this reduction in self-control extends to cyberspace, fostering a propensity for online disinhibition (Gokalp and Haktanir, 2024), which ultimately precipitates online flaming, such as online insults and malicious comments (Kurek et al., 2019).

### Gender difference

A multigroup comparison revealed cross-gender invariance in the overall model, indicating that the pathways from interparental conflict to online flaming are equivalent for boys and girls. This cross-gender consistency may stem from similar network availability and shared security demands. In the anonymous, invisible online space, people of both genders regard the internet as a safe outlet to vent emotions, ease stress, and release aggression. Despite the overall invariance, a few individual paths showed gender differences. Pairwise comparisons indicated gender differences in the direct effect of interparental conflict on psychological stress. This result is consistent with a prior study (Davies and Cummings, 1994), which found that when facing interparental conflict, male adolescents tend to experience threat, whereas female adolescents tend to self-blame, and that males experience greater psychological stress. Supporting Male Role Norm Theory (Oransky and Fisher, 2009), Chinese cultural norms encourage males to suppress emotions to maintain masculinity, leading to higher stress. Females, who are more expressive, experience comparatively less stress (Eagly and Wood, 2012; Wang et al., 2022). The mediating effect of toxic online disinhibition between interparental conflict and online flaming revealed a marginally significant gender difference. The findings are consistent with Male Role Norm Theory and Social Role Theory (Oransky and Fisher, 2009; Eagly and Wood, 2012): males exhibit greater toxic online disinhibition and, consequently, more online flaming, as such aggression serves as a masculine display of dominance. In contrast, females conditioned to exhibit kindness and gentleness demonstrate lower toxic online disinhibition, and thus participate in less online flaming.

### Practical implications

This study provides practical implications for preventing and intervening in online flaming among junior high school students. First, interparental conflict has been confirmed as a risk factor for online flaming, indicating that interventions targeting interparental conflict can effectively reduce such behavior in adolescents. These interventions can be implemented through both proactive prevention and reactive coping. Proactive programs, such as the “Family Foundations” initiative, enhance coparenting quality to alleviate emotional problems (e.g., depression and anxiety) in couples and improve parenting practices, thereby reducing behavioral and emotional problems in children (Feinberg, 2002; Feinberg et al., 2009). Reactive programs, such as the “Family Communication” intervention, focus on strengthening constructive components and curbing destructive components in both marital and parent–child conflicts, to enhance adolescents' emotional security and adaptive capacity (Miller-Graff et al., 2016). Second, online disinhibition mediates the relationship between interparental conflict and online flaming. Because of the online disinhibition effect, adolescents may proactively engage in verbal attacks or reactively retaliate with insults. Concerning proactive aggressive behavior, teachers and parents should guide adolescents to recognize that cyberspace is not a lawless realm, that personal ethics must be upheld even in online environments, and that aggressive words and actions can cause serious distress to others. With regard to reactive aggressive behavior, adolescents need to understand that deliberate provocateurs often aim to elicit abusive or aggressive responses, and that they can directly report such situations. Meanwhile, instant messaging software should automatically detect and filter vulgar and harmful speech to reduce the occurrence of reactive aggression. In addition, the government should strengthen public awareness campaigns to ensure that adolescents are aware that online expressions are tied to real-name identification and that regulators can trace individual identities through technical means such as IP addresses, thus curbing disinhibited impulsive speech and behavior. Third, this study found that psychological stress not only directly mediates the effect of interparental conflict on online flaming but also exerts an indirect effect through online disinhibition, forming a chain mediating pathway of “interparental conflict → psychological stress → online disinhibition → online flaming.” Accordingly, parents and teachers should closely monitor adolescents' psychological state; once elevated stress levels are detected, they should provide timely psychological counseling, teach effective stress-regulation skills, or assist them in seeking help from mental health professionals. Reducing psychological stress in this way can interrupt the subsequent chain of disinhibition and aggressive behavior. These inferences suggest that distal family risk factors transmit their influence through proximal intra-individual implicit processes, layer by layer, constituting the generative mechanism of online flaming. Hence, practical intervention should simultaneously focus on conflict resolution, stress management, and environmental constraints on disinhibition, so as to form a comprehensive protective chain.

### Research shortcomings and prospects

The cross-sectional design of this study did not elucidate the causal relationship between the research variables; future studies may employ longitudinal designs to further investigate this association. Secondly, the sample was limited to students in Grades 7–9, restricting the developmental scope. Future studies should include a broader grade range to enhance external validity. Third, as the findings were obtained using self-report measures, they may represent subjective views shaped by participants' cognitive biases, motivational states, and memory constraints, rather than accurately reflecting objective behavioral outcomes. Future research could mitigate this issue by employing anonymous response protocols and methodological triangulation—such as direct behavioral observations and comprehensive interviews—to validate and enhance self-reported data. Fourth, the study used convenience sampling. As a non-probability method, it is prone to sampling bias, which limits the generalizability of the findings. Future research should employ a probability-based design—for example, cluster sampling with a well-defined sampling frame of secondary schools. This would effectively improve the sample's representativeness and the external validity of the conclusions. Finally, the present study examined the mediating mechanism of psychological stress and online disinhibition between interparental conflict and online flaming, but the process is probably affected by parenting. For example, positive parenting style (Warmth, Behavioral Control, Autonomy Granting, and Authoritative Parenting) is significantly negatively associated with internalizing and externalizing problems, which may further reduce the effect of interparental conflict on other variables; however, negative parenting style (Harsh Control, Psychological Control, Authoritarian Parenting, Permissive Parenting, and Neglectful Parenting) shows the opposite association (Pinquart, 2017a,b). Future research should investigate how varying parenting styles might mitigate or exacerbate the stress, online disinhibition, and online flaming that arise from interparental conflict.

## Conclusion

Online flaming is a prevalent risk manifestation during adolescence that has drawn sustained attention in the field of psychology. However, few studies have systematically examined the predictive role of interparental conflict on such behavior while simultaneously uncovering the underlying mediating mechanisms and gender differences. After controlling for age and gender, the present study found that interparental conflict positively predicted online flaming, and that psychological stress, toxic online disinhibition, and benign online disinhibition all mediated this relationship. More importantly, psychological stress further contributed to toxic and benign online disinhibition, respectively, thereby forming serial mediating pathways of “interparental conflict → psychological stress → online disinhibition → online flaming.” Multi-group analysis revealed no significant gender differences in the overall model; however, pairwise comparisons further indicated gender-specific variations in two pathways: the direct effect of interparental conflict on psychological stress was stronger in males, and the mediating effect of toxic online disinhibition in the interparental conflict–online flaming link was also more pronounced in males. These results suggest that adolescents who experience interparental conflict tend to endure greater psychological stress and exhibit stronger disinhibition tendencies in the online environment (especially toxic disinhibition), which in turn leads to more frequent online flaming. Accordingly, whether by reducing interparental conflict at its source or by intervening in subsequent psychological stress and online disinhibition, it may be possible to effectively reduce the occurrence of such behavior. However, limited by the cross-sectional design, although all the direct and indirect associations reached statistical significance, they cannot support causal inferences, nor can they establish the temporal order among the variables.

## Data Availability

The raw data supporting the conclusions of this article will be made available by the authors, without undue reservation.
